# Whole-genome variance components linkage analysis using single-nucleotide polymorphisms versus microsatellites on quantitative traits of derived phenotypes from factor analysis of electroencephalogram waves

**DOI:** 10.1186/1471-2156-6-S1-S15

**Published:** 2005-12-30

**Authors:** Yi Yu, Yan Meng, Qianli Ma, John Farrell, Lindsay A Farrer, Marsha A Wilcox

**Affiliations:** 1 Genetics Program, Department of Medicine, Boston University School of Medicine, Boston, Massachusetts 02118, USA

## Abstract

Alcohol dependence is a serious public health problem. We studied data from families participating in the Collaborative Study on the Genetics of Alcoholism (COGA) and made available to participants in the Genetic Analysis Workshop 14 (GAW14) in order to search for genes predisposing to alcohol dependence. Using factor analysis, we identified four factors (F1, F2, F3, F4) related to the electroencephalogram traits. We conducted variance components linkage analysis with each of the factors. Our results using the Affymetrix single-nucleotide polymorphism dataset showed significant evidence for a novel linkage of F3 (factor comprised of the three midline channel EEG measures from the target case of the Visual Oddball experiment ttdt2, 3, 4) to chromosome 18 (LOD = 3.45). This finding was confirmed by analyses of the microsatellite data (LOD = 2.73) and Illumina SNP data (LOD = 3.30). We also demonstrated that, in a sample like the COGA data, a dense single-nucleotide polymorphism map provides better linkage signals than low-resolution microsatellite map with quantitative traits.

## Background

Alcoholism is a complex disorder involving multiple genes likely interacting with one another and environmental factors. Quantitative endophenotypes, such as electroencephalogram (EEG) measurements, have been suggested as better indices of alcoholism susceptibility than the customary dichotomous affection status [1,2]. EGG data defined by different experimental designs were available to participants in Genetic Analysis Workshop 14 (GAW14). Since EEG phenotypes are correlated, it is likely that a smaller number of underlying dimensions contribute to the variance of these EEG phenotypes. Our aim was to identify the underlying factors for the EEG phenotypes and search for genes influencing the derived factors and increasing the risk of alcohol dependence.

## Methods

### Phenotypes and factor analysis

We conducted a principal components analysis using the 12 EEG measures (ttth1-ttth4, ttdt1-ttdt4, and ntth1-ntth4). EEG measures from the Visual Oddball experiment were represented as four letters followed by a number (ttth1-ttth4, ttdt1-ttdt4, and ntth1-ntth4). The four letters denote different experiment conditions: ttth_ contain extracted measures from the target case correspond to the 'late' time window, which is set at 300 to 700 ms following stimulus presentation (bounding the visual P3 event), and the theta band power (3 to 7 Hz). ttdt_ contain extracted measures which the delta band power is 1 to 2.5 Hz with other conditions same as ttth_. The fields labeled ntth_ contain extracted measures from the non-target case correspond to the 'early' time window, which is set at 100 to 300 ms following stimulus presentation, and the theta band power (3 to 7 Hz). The number following the four letters denotes the locations of the 4 electrode placements: 1 – FP1 (far frontal left side channel), 2 – FZ (frontal midline channel), 3 – CZ (central midline channel), 4 – PZ (parietal midline channel).

This was followed by a common factor analysis in order to identify the underlying dimensions measured by the EEG data. We examined each of the phenotypes for normality before including it in the analysis. In the common factor model, each new phenotype is expressed as a linear combination of the original variables. The relationship of factors to the EEG phenotypes is reflected by factor loadings. The contribution of each factor to the set of variables is evaluated by eigenvalues. Based upon the distribution of the eigenvalues and the composition of the factors, we retained four factors. This solution accounted for 88% of the total variance. We used an oblique rotation of the factor solution. Factor scores were obtained using PROC FACTOR implemented in SAS (SAS version 8; SAS, Cary, NC). We treated each of the four factor scores as a new derived quantitative trait.

### Map construction

Quantitative data usually provide more statistical power than a binary affection status. However, using the quantitative traits alone may still not be powerful enough to identify disease susceptibility genes for complex traits. Kruglyak predicted that using single-nucleotide polymorphism (SNPs) with a heterozygosity of 0.50 and approximately two to three times the density of the current microsatellite marker sets would achieve a similar result in linkage analysis as a genome scan with microsatellite markers [3]. Recently John et al. conducted a whole-genome scan using SNPs [4]. Their results showed that SNPs provided significantly higher information content than microsatellites and allowed loci to be defined more precisely. We hypothesized that there would also be higher information content, and better linkage signals for SNPs compared with microsatellites for quantitative traits. We carried out a whole-genome screen using 143 families from the Collaborative Study on the Genetics of Alcoholism (COGA) with four empirically derived quantitative traits (factor scores based upon the EEG data). Reformatted clean genotype data were provided by the COGA study, including 11,120 SNPs generated by Affymetrix GeneChip Mapping 10 K Array, 4,720 SNPs generated by Illumina, and 328 microsatellite markers spaced at 10-cM intervals across the genome. Both microsatellite and SNP genetic map positions were interpolated based upon the deCode genetic framework map, calculated based on their physical positions. Physical positions of SNPs were obtained from the NCBI database (release 34.3). SNPs with multiple physical map positions were dropped from the genetic map. All initial linkage analysis was performed using this adjusted map.

### Linkage disequilibrium (LD)

Because linkage analysis algorithms assume linkage equilibrium between all markers, strong LD between SNPs may exaggerate the significance level of linkage and thus generate false positive results [5]. So we kept only one tag SNP in each haplotype block (SNPs in strong LD). The pairwise LD statistics D' and *r*^2 ^were calculated for all SNPs by HAPLOVIEW (v3.0) [6]. Haplotype blocks were defined as regions over which a very small proportion (<5%) of comparisons among informative SNP pairs showed strong evidence of historical recombination [7].

### Linkage analysis

We performed variance components analysis for each factor by using SOLAR (v2.13) [8]. In variance components analysis, the total variance of each trait was decomposed into several sources by the following equation:

Ω = Πσ^2^_*q *_+ 2Φσ^2^_*g *_+ Iσ^2^_*e*_,

where Ω is the covariance matrix for a pedigree, Π is a matrix with elements π_*qij*_, which is the expected proportion of genes two individuals share as identical by descent (IBD) at specific chromosomal location, Φ is the kinship matrix, I is the identity matrix, σ^2^_*q *_is the variance component corresponding to the additive genetic effects from the major locus, σ^2^_*g *_is the variance component corresponding to the polygenic effects, and σ^2^_*e *_is the variance component corresponding to the environmental effects. The variance components analysis tested the null hypothesis that the additive genetic variance caused by the major quantitative trait locus (QTL) for a given trait equals zero (H_0_: σ^2^_*q *_= 0, or no linkage). The hypothesis testing was conducted by comparing the maximum likelihood of a restricted model in which σ^2^_*q *_was constrained to zero with a more general model in which σ^2^_*q *_was estimated, using the likelihood ratio test. Twice the difference of the natural logarithm likelihoods of the two models yields a test statistic that is asymptotically distributed as a 50/50 mixture of a χ^2 ^and a point mass of zero. The log_10 _of the likelihood ratio between the two models yields a LOD score that is equivalent to the classical LOD score of linkage analysis [8]. The IBD matrix, multipoint IBD matrix, and heritability (*h*^2^) for each factor were estimated using SOLAR.

## Results

EEG measures and loadings on each of the four factors (F1, F2, F3, F4) obtained from factor analysis are shown in Table 1. Two alcoholism classifications were provided in the COGA data. ALDX1 was based on the DSM-III-R and the Feighner criteria. ALDX2 was defined by the DSM IV criteria. Table 2 shows the results of an analysis of variance (ANOVA) comparing the factor scores for affection status groups defined by ALDX1 and ALDX2. F3 (the three midline channel EEG measures from the target case of the Visual Oddball experiment ttdt2, 3, 4) was significant in both ALDX1 and ALDX2, indicating subjects with different affection status for alcohol dependence have different F3. Post-hoc comparisons using the Bonferroni method show that F3 was significantly higher in the unaffected with some symptoms group than in the affected group (*p *< 0.05). Similar patterns were seen in ttdt3 and ttdt4.

**Figure 1:**
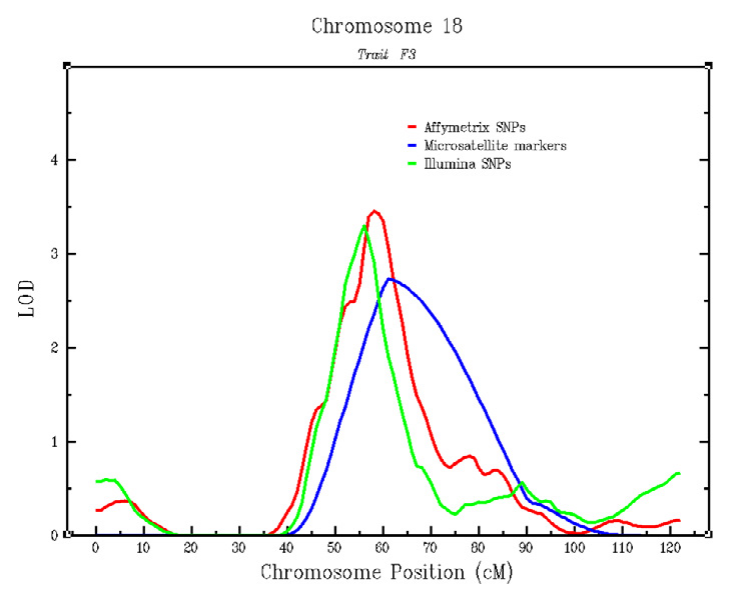
**Multipoint LOD scores on chromosome 18 for trait F3**. Multipoint LOD scores on chromosome 18 for trait F3 respectively using Affymatrix SNPs (red), Illumina SNPs (green) and microsatellites (blue) datasets.

**Figure 2:**
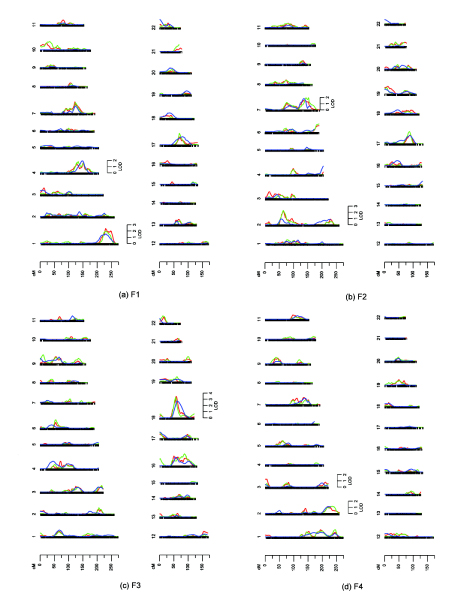
**Genome-wide scans for each Factors in each datasets**. Genome-wide scans for F1(a), F2 (b), F3 (c) and F4 (d) as quantitative traits, using Affymatrix SNPs (red), Illumina SNPs (green) and microsatellites (blue) datasets.

We examined the heritability of each of the quantitative traits. Heritability for F1 (34.5 ± 6.6), F2 (32.1 ± 5.9), F3 (30.7 ± 6.2), and F4 (30.8 ± 6.7) was all significant (*p *< 0.001). We found significant evidence of linkage for F3 to chromosome 18 (LOD = 3.45 at 58 cM) in the Affymetrix SNP dataset. We had similar findings in the microsatellite (LOD = 2.73 at 61 cM) and Illumina SNP dataset (LOD = 3.30 at 56 cM) (Figure 1). Linkage peaks (LOD > 1.0) for each of the four factors are presented in Table 3. All genome scan results for each factor in each genotype dataset are shown in Figure 2.

## Discussion

In the present study, our work suggests that there are four factors underlying the EEG measures. Among the four factors, factor 3 (F3), representing the midline measures (EEG ttdt2, 3, 4), was significantly different between affection status groups as defined by both ALDX1 and ALDX2.

We found a novel genetic locus with significant evidence of linkage to F3 (EEG ttdt2, 3, 4) on chromosome 18, indicating this region (18q12.1-12.3) may harbor a gene that confers liability for alcohol dependence. A search of genome databases revealed a potential candidate gene SYT4 located in the genetic locus on 18q12.3 where we found significant linkage. Synaptotagmin-4, encoded by SYT4, may play an important role in the Ca^2+^-dependent release of neurotransmitters and neuropeptides from the presynaptic nerve terminal. SYT4 expression was only detected in the brain, and was highest in the hippocampus [9]. An animal model showed that Syt4 mutant mice displayed impaired social transmission of food preference and disrupted contextual fear conditioning [9]. Based on the evidence from our linkage study and the gene function revealed by other studies, SYT4 may be a determinant of alcohol dependence and is a candidate for further study.

By using the SNPs in the genome-wide linkage analysis we observed a higher LOD score than using the microsatellite markers. The peak of linkage was also sharper for the SNPs with a smaller confidence interval than for the microsatellite markers.

## Conclusion

In this study, our results from both SNPs and microsatellites suggest that there is a strong linkage of F3, which mostly consists of ttdt2, ttdt3 and ttdt4, to chromosome 18. We demonstrated that, in a sample like the COGA data, a dense SNP map with a quantitative trait could provide better linkage signals than low-resolution microsatellite scan for linkage analysis, and would also help define the peak of linkage more precisely.

## Abbreviations

ANOVA: Analysis of variance

COGA: Collaborative Study on the Genetics of Alcoholism

EEG: Electroencephalogram

GAW: Genetic Analysis Workshop

IBD: Identical by descent

LD: Linkage disequilibrium

QTL: Quantitative trait locus

SNP: Single-nucleotide polymorphism

## Authors' contributions

YY participated in the design of the study, performed the statistical analysis and drafted the manuscript. YM, QM, JF, and LAF participated in its design and coordination. MAW participated in the design of the study and helped to draft the manuscript. All authors read and approved the final manuscript.

## Figures and Tables

**Table 1:** Factor loadings pattern – oblique rotation

**EEG phenotypes**	Factor loadings*			
	
	F1	F2	F3	F4
ntth3	**0.90**	0.11	0.07	-0.08
ntth4	**0.86**	0.10	0.07	-0.17
ntth2	**0.83**	0.02	-0.01	0.21
ntth1	**0.65**	-0.11	-0.09	**0.52**
ttth3	0.05	**0.92**	0.06	-0.01
ttth4	0.09	**0.87**	0.08	-0.10
ttth2	0.05	**0.75**	-0.02	0.31
ttdt3	0.04	0.02	**0.89**	0.06
ttdt4	0.07	0.10	**0.81**	-0.05
ttdt2	-0.06	-0.01	**0.66**	**0.42**
ttdt1	-0.07	-0.10	0.24	**0.72**
ttth1	0.04	0.36	-0.09	**0.71**

*Loadings greater than an absolute value of 0.40 are shown in bold.

**Table 2:** Relationship between factors and affected status

**Factors**	***p*-value (ALDX1)**	***p*-value (ALDX2)**
F1	0.1603	0.0502
F2	0.1515	0.0271
F3	0.0018	0.0431
F4	0.4099	0.4800

**Table 3:** Linkage peaks found in genome scans

**Trait**	**Location**	**MS**	**Illumina**	**Affymetrix**
F1	1q42	1.51	1.93	2.40
F1	4q31	1.91	1.79	1.58
F2	2p22	0.92	2.30	2.21
F2	7q33	1.81	1.92	1.50
F3	18q12	2.73	3.30	3.45
F4	2q36	1.13	1.19	1.12

## References

[B1] Almasy L (2003). Quantitative risk factors as indices of alcoholism susceptibility. Ann Med.

[B2] Porjesz B, Begleiter H (2003). Alcoholism and human electrophysiology. Alcohol Res Health.

[B3] Kruglyak L (1997). The use of a genetic map of biallelic markers in linkage studies. Nat Genet.

[B4] John S, Shephard N, Liu G, Zeggini E, Cao M, Chen W, Vasavda N, Mills T, Barton A, Hinks, Eyre S, Jones KW, Ollier W, Silman A, Gibson N, Worthington J, Kennedy GC (2004). Whole-genome scan, in a complex disease, using 11,245 single-nucleotide polymorphisms: comparison with microsatellites. Am J Hum Genet.

[B5] Schaid DJ, McDonnell SK, Wang L, Cunningham JM, Thibodeau SN (2002). Caution on pedigree haplotype inference with software that assumes linkage equilibrium. Am J Hum Genet.

[B6] Barrett JC, Fry B, Maller J, Daly MJ (2005). Haploview: analysis and visualization of LD and haplotype maps. Bioinformatics.

[B7] Gabriel SB, Schaffner SF, Nguyen H, Moore JM, Roy J, Blumenstiel B, Higgins J, DeFelice M, Lochner A, Faggart M, Liu-Cordero SN, Rotimi C, Adeyemo A, Cooper R, Ward R, Lander ES, Daly MJ, Altshuler D (2002). The structure of haplotype blocks in the human genome. Science.

[B8] Almasy L, Blangero J (1998). Multipoint quantitative-trait linkage analysis in general pedigrees. Am J Hum Genet.

[B9] Ferguson GD, Chen XN, Korenberg JR, Herschman HR (2000). The human synaptotagmin IV gene defines an evolutionary break point between syntenic mouse and human chromosome regions but retains ligand inducibility and tissue specificity. J Biol Chem.

